# Headaches*Read before the West End Medical Society, of Louisville, Ky.

**Published:** 1914-05

**Authors:** Ernest H. Koch

**Affiliations:** Louisville, Kentucky


### Headaches.*

BY ERNEST H. KOCH, M. D., LOUISVILLE, KENTUCKY.

In presenting the subject of headache for consideration, the writer recognizes he is suggesting a theme about which authors have disputed for years. While, generally speaking, headache is considered symptomatic, yet the disorder may be primary, and much confusion has arisen in attempting to clearly distinguish between the different varieties. The fact remains, however, that the patient usually complains of pain somewhere about the head, and this the physician is asked to immediately relieve, it matters not what may be the underlying cause.

There are so many causes of headache that it would be impossible in a short paper to discuss each one separately. The writer will therefore give a general classification of the disorder under five headings, as to location and causation, then briefly mention some of the more common forms usually observed.

(1) Unilateral head pain may depend upon hysteria, dysmenorrhea, trigeminal neuralgia, migraine, disease of the ear, disease of bone, cancer of the tongue, lithemia, weak or painful vision.

(2) Vertex pain may be due to anemia, chlorosis, hysteria,

*Read before the West End Medical Society, of Louisville, Ky.

neurasthenia, epilepsy, disease of the uterus, the ovaries or the bladder.

(3) Frontal and temporal pain may be the result of anemia, neurasthenia, nephritis, uremia, dyspepsia, constipation, myalgia, rheumatism of the scalp, lithemia, eyestrain, glaucoma, iritis, disease of or foreign bodies in the nasopharynx, disease of the frontal sinus, syphilitic nodes or periostitis, and neuralgia of the fifth nerve.

(4) Occipital and cervical pain may be caused by epilepsy, neurasthenia, spinal irritation, meningitis, cerebellar tumors, or lesions, dyspepsia and constipation, disease of the cervical vertebræ, cervico-occipital myalgia and neuralgia, adenoids, middle ear disease, eyestrain, carious teeth, uterine disease, syphilis, nasopharyngeal disease, rheumatism, disease of the sphenoidal sinus, nephritis and uremia.

(5) Pain in the eyeballs may be the result of migraine, neuralgia of the fifth nerve, ophthalmoplegia interna, coryza, inflammation of the conjunctiva, iris or cornea, glaucoma, weak or painful vision.

The average physician not infrequently encounters the so-called ear disease headache, which is oftentimes combined with earache or mastoid pain. In such cases the pain is parietal, oftentimes widespreading, and is usually increased by jaw movements. Pain on pressure behind the ear indicates that extension to the mastoid has ensued. Abscess of the brain may follow disease of the middle ear or labyrinth. The treatment is usually paliative, when the trouble is purely catarrhal or serous—heat, moist or dry, belladonna, aconite—or surgical where pus is present.

The various toxemias are common causes of headache. In lead poisoning, which is usually accompanied by severe nephritis, the headache is diffuse, in mild cases being described as a pressure or heavy feeling. In the more severe forms, however, the headache is extreme, associated with signs of mental hebetude, and at times with convulsive movements. The characteristic blue gum line, colic, the presence of albumin in the urine and other symptoms make the diagnosis certain. The therapy is directed toward prevention principally by cleanliness, and elimination by salines.

In chronic nicotine poisoning, especially excessive cigarette smoking, occipital headache is frequently observed, and is commonly associated with pressure symptoms. Like other toxic headaches, this form results from nerve blood pressure changes.

Acute alcoholism and acute morphinism are oftentimes associated with severe frontal headache. In the former an intense hyperesthesia of the scalp is quite characteristic. In the latter a basal or occipital headache is frequently accompanied by itching of the skin of the body. If the toxic materials are still operative, prompt enuresis and catharsis are indicated. Coffee or caffein, in combination with the bromides or coal tar products, will be found beneficial. Gastric lavage is helpful.

In nephritic headache the pain is usually heavy rather than sharp, the patient complaining of pressure or heaviness, more rarely acute pain, in the forehead. Albumin in the urine, diminished urea excretion, high blood pressure and other signs of uremic poisoning confirm the diagnosis. The treatment depends upon the form of nephritis which may be present.

Diabetic headache occurs as a diffuse pressure, heaviness, or neuralgia. Preceding diabetic coma the head pain becomes more severe. Sugar in the urine, thirst, itching of the skin, etc., establish the diagnosis. The therapy is that of diabetes in general.

In anemia and chlorosis the headache is intensely severe and continuous, and pain involves the entire head. The diagnosis is established chiefly by the color and modified physical condition of the patient, which may be confirmed by blood examination. The iron salts, arsenic, proper food and sanitation will usually be sufficient to afford relief. In cases of this character preparations containing acetanilid do harm, because they interfere with the iron oxygen interaction in the red blood cells, thus diminishing the functional capacity of the already reduced iron content of the cell.

Gastrointestinal headache is frequently reflex in character, such as that induced by an "empty" or "hungry" stomach. Similarly various forms of indigestion are accompanied by dull or severe headache, chiefly frontal. Most migraine attacks have definite gastrointestinal disturbances as forerunners. The headache of constipation is frequently a sense of pressure oftentimes relieved by a free stool.

Headache is commonly an obstinate symptom of the infectious diseases, particularly influenza, in which the pain is usually occipital. It is low in grade, rarely advancing to the sharp pain of neuralgia. It develops after the slightest mental effort, such as reading a few lines of print, etc. There may be no other definite symptoms, and the patient does not suffer at night, nor when walking. Such a headache may persist for weeks or months. The proper treatment is massage of the back of the head, hot baths, frequent feeding, two or three weeks rest cure. If the patient is unable to rest in bed, moderate exercise may be permitted. Free catharsis is desirable. Combinations of bromides and analgesics may at times be necessary, but opiates are to be avoided.—*The American Practitioner.*

